# Bridging the gap between CBME in theory and practice: the role of a teacher community

**DOI:** 10.1007/s40037-014-0147-7

**Published:** 2014-12-09

**Authors:** Ale Gercama, Thea van Lankveld, Johanna Kleinveld, Gerda Croiset, Rashmi Kusurkar

**Affiliations:** 1 VUmc School of Medical Sciences, Institute of Education and Training, VU University Medical Center Amsterdam, Henninkstraat 20, 1435 HM Rijsenhout, The Netherlands; 2 Department of Research and Theory in Education, Faculty of Psychology and Education, VU University Amsterdam, Amsterdam, The Netherlands; 3 LEARN! Research Institute for Learning and Education, VU University Amsterdam, Amsterdam, The Netherlands

**Keywords:** Teacher community, Curricular reform, Organizational learning

## Abstract

The success of curricular reforms is dependent on the teachers who put the reforms into practice. In medical education, clinicians as teachers are important in this endeavour and the educational organization can learn from their efforts. The ‘Vygotsky space’ construct may help to analyze the learning process between teachers and organization. A case study is presented that shows how a teaching community acted as an enabler in this learning process.

## The problem

Curricular reforms in medical education have focused on changing teacher-centred learning to student-centred learning and self-directed learning [1, 2]. The teacher’s role in this process is that of a guide and facilitator and not merely that of a transmitter of knowledge or a content expert [3]. In these curriculum reforms, it sometimes happens that the teachers who actually implement the curricular reforms are not the decision-makers regarding the content of these reforms [4]. Many medical education institutes or organizations have separate centres or departments of education which are responsible for the content and quality of the curriculum and faculty development [5]. Thus, curricular reforms are requisitioned by the educational organization or institute and are designed by curriculum committees. There can be a wide gap between the educational organization and the teachers, which can lead to failure of implementation of curricular reforms. Apart from curriculum mapping, as recommended by Harden [6], specific ways of bridging this gap have not been addressed sufficiently in the literature.

In 2005, the VUmc School of Medical Sciences (Amsterdam, The Netherlands) introduced a new undergraduate curriculum within its Bachelor/Master’s structure, based on competency-based medical education (CBME). The events that followed brought to light a gap between the objectives of the responsible educational organization and the experiences of the teachers. In this article, when we talk about educational organization we mean the organization that develops and organizes the curriculum, excluding those who do the actual teaching.

## The question

The question addressed was: How can the interaction between the educational organization and teachers be facilitated to make curricular reform successful?

In order to examine this question we used a framework of organizational learning as suggested by Peck et al. [7] for understanding the relations between learning at the level of the teachers and learning at the level of the organization. Peck made use of the Harré model based on the socio-cultural learning theory of Vygotsky, the ‘Vygotsky space’ [5].

**Fig. 1:**
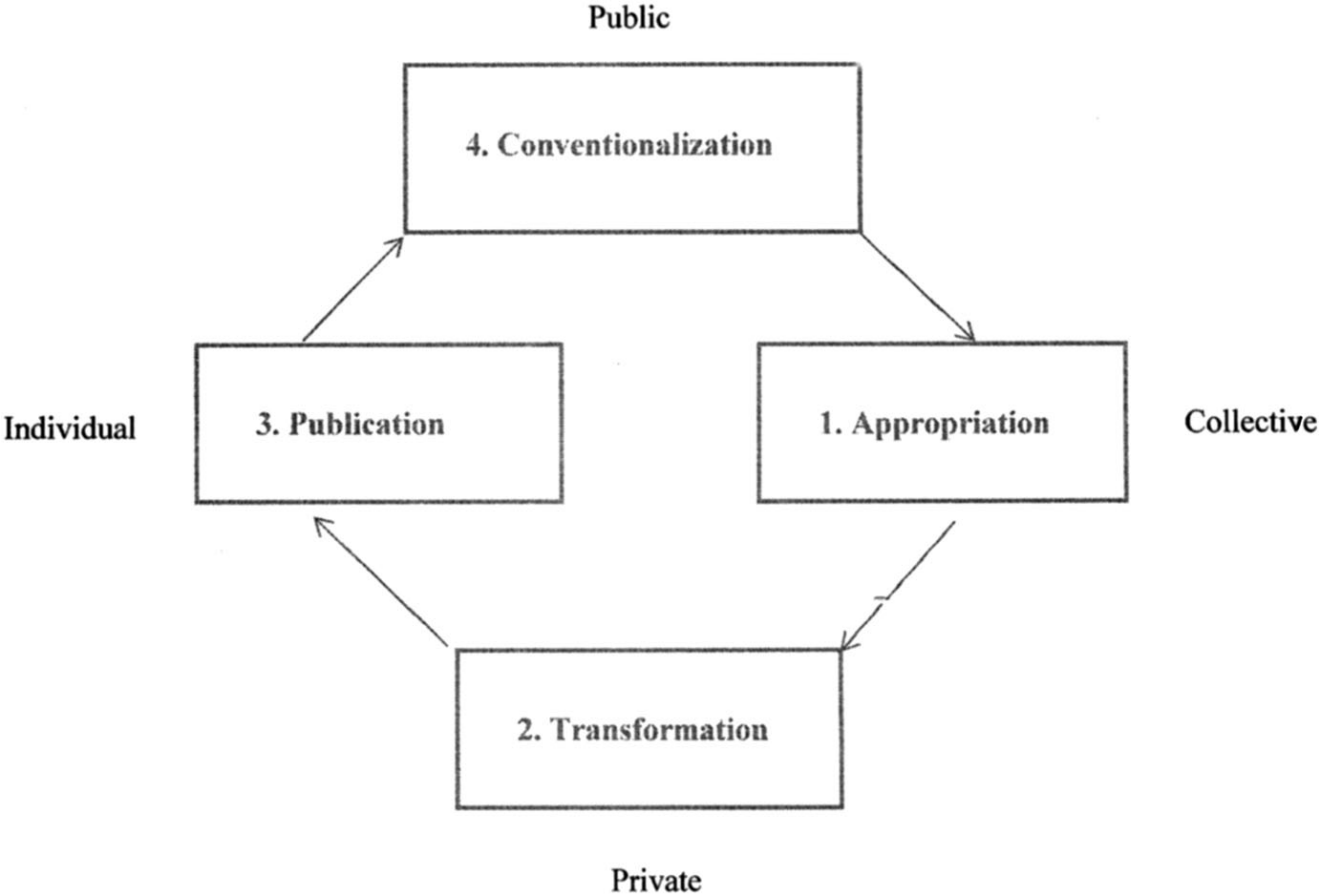
The Vygotsky space with its four dimensions and phases (adapted from Peck et al. [7].)

The Vygotsky space represents learning in terms of relationships between collective and individual actions and between public and private settings. The interactions between these two dimensions are then conceptualised as four phases of a process (Fig. 1):

1. *Appropriation* is the phase in which new practices are taken up by individuals. This phase is preceded by a ‘disturbance’ in the organizational equilibrium, such as a change in the policy of the organization.
2. *Transformation* is the phase in which these new practices are adapted and changed in the context of individual needs and uses.
3. *Publication* is the phase in which these adapted practices are externalized to others.
4. *Conventionalization* is the phase in which these practices are adopted by others, which could be individuals or the organization.

When we apply the Vygotsky space process to medical education, the ‘disturbance’ is the introduction by the educational organization of new educational objectives, *appropriation* is the phase in which the teachers are instructed to take up new functions such as instructor, coach and critical observer in order to achieve these objectives, *transformation* is the phase in which the teachers adapt these functions to their own ideas and capabilities, *publication* is the phase in which these individual adaptations are being discussed with the organization and *conventionalization* is the phase in which the organization adopts the results of these discussions and uses them to improve the teaching programme.

A case study will be presented in which the interplay between the educational organization and the teachers was analyzed with the help of the Vygotsky space and in which a teacher community proved to be a means to bridge the aforementioned gap between the two.

## The case study

### Disturbance and appropriation

In the first Master year, students had difficulty in applying their medical knowledge during clerkships. To prepare students better, the curriculum committee organized a new course in the two semesters of the third Bachelor year (B3). The main objectives were the improvement of students’ abilities in clinical reasoning and critical appraisal of clinical literature. In this new course, groups of 12 students gather weekly during a semester with an experienced clinician as their teacher for a brainstorm session on clinical problems. Questions that are raised during the brainstorm are worked out by the students during the rest of the week and at the end of the week remaining questions can be presented by them in ‘meet the expert’ sessions. In addition, the students review the medical literature in their group and report on visits they make to departments in the academic hospital. The teacher/clinician is expected to use these learning opportunities as a means to prepare the students for medical practice. This means that the teacher should be able to give instruction in clinical reasoning and critical reading of clinical literature, to observe student performance critically and to stimulate students’ reflections on it. The medical content is arranged in integrated themes, e.g. ‘inflammation’ (four themes in each semester). The curriculum committee, consisting of course organizers, takes care of both development and organization of the programme with each course organizer responsible for one theme.

Teachers showed great variety both in clinical background and experience in clinical reasoning with students. Before starting their task, they were invited for a half day introduction to get familiar with the method and goals of the course. In addition they were invited to 1 h briefings before a new theme started.

### Transformation

The teachers of the new course were invited to a ‘teacher community’ based on the concept of ‘communities of practice’ [8]. Teacher communities are groups of teachers that share a concern or passion for teaching and that meet regularly on a voluntary basis to discuss their teaching practices and find solutions to the problems they experience [9]. During a semester course, members of the community met monthly during lunchtime. The meetings were chaired by a facilitator. Notes were made of each meeting and a summary of the results was forwarded to the curriculum committee.

Eight (out of 30) teachers took part in the teacher community. They said they had encountered difficulties in engaging the students. They said the students were reluctant to mobilize their prior knowledge, partly due to lack of preparation and partly due to greater interest in ‘new’ knowledge. Also, the case histories presented were often not helpful, in the eyes of the teachers. They were either too complicated or followed by questions that did not encourage the students to work independently in the period between the group session and the ‘meet the expert’ session. At this *transformation phase*, the teachers had tried to overcome these problems with actions such as taking more initiative, giving examples, asking questions or letting the students take the initiative to discuss their ‘lines of clinical reasoning’ afterwards. They adapted the original ideas of the course in the context of their own needs and insights.

### Publication

In the teacher community, the teachers discussed their experiences and adapted practices. In doing so, they realized that the students were habituated to an extrinsically motivated test-driven learning strategy that is primarily geared to the acquisition of ‘new’ knowledge (through a surface learning approach) rather than a more intrinsically motivated strategy which would integrate the new knowledge in the already existing knowledge in order to solve clinical problems (deep learning approach) [2]. The teachers felt the need to discuss with students why a change was necessary and how to accomplish it.

In the community meetings, the teachers discussed different techniques to promote the level of clinical reasoning in relation to the situations in which they were used and the results obtained. This led to a better understanding about how and when to use these techniques. The facilitator of the community gave feedback to the curriculum committee regarding the need for basic information around key features as an aid to clinicians who are not experts in the particular case. The facilitator also communicated the need to formulate strategies how to facilitate the mobilization of prior knowledge in each case history, together with suggestions from the community on how to realize these needs in a practical way.

### Conventionalization

The phase in which adapted practices are adopted by others already started during the course. One of the teachers engaged in the educational organization as a course organizer, used the results of the *publication phase* to construct the cases and the set-up of the ‘meet the expert’ sessions later in the year. The feedback given was implemented successfully by the course organizers, and a list of ‘Tips and Tricks for Tutors’ derived from the teacher communities was made available to all teacher/clinicians.

## Discussion and conclusion

Analysis of the learning process between teachers and the educational organization with the help of the Vygotsky space model, shows that when teachers experience a gap between the objectives of the course and the learning activities of the students, a community helps teachers to discuss and summarize their *transformation* activities and to make them ready for *publication and conventionalization*. In doing so, the community may function as an enabler to take an initiative or change to a successful level and may strengthen the evaluation loop of the course. Whether this will happen depends on the impact of the community on the whole group of teachers/clinicians involved in the course and the readiness of the educational organization to consider its results for implementation. In our case the teachers who did not take part in the community appreciated the role of the community in clarifying the amount of freedom they had in working towards the goals of the course and how they could make use of the ‘Tips and Tricks for Tutors’ in doing so. The educational organization used the results as an addition to the formal evaluation procedure of the course and to adjust the introductory course and the programme of the briefings to the needs of the teachers/clinicians.
